# Visual masking by transcranial magnetic stimulation in the first 80 milliseconds

**DOI:** 10.2478/v10053-008-0023-2

**Published:** 2008-07-15

**Authors:** Thomas Kammer

**Affiliations:** Department of Psychiatry, University of Ulm, Germany

**Keywords:** visual cortex, masking, feedforward, re-entrant, magnetic stimulation

## Abstract

Stimulation of the occipital cortex with transcranial magnetic stimulation (TMS) can interfere with visual processing and may cause masking comparable to visual masking. The effect is most pronounced when the TMS pulse is delivered with stimulus onset asynchronies (SOAs) of 80-100 ms. In a few experiments a second time window of TMS-induced visual masking has been identified with its maximum around an SOA of 40 ms. The existence of two masking windows has been taken as evidence for two distinct visual processes taking place in V1: an early feedforward component and a later re-entrant feedback component. The evidence for the existence of two separate TMS time windows is reviewed. The early time window was not reproducible in all the attempts to characterize TMS masking effects. Interindividual anatomical differences in the location of V1 might contribute to the heterogeneous results.

## Introduction

The dynamics of early visual processing are still not completely understood. Lamme et al. (Lamme, Supèr, & Spekreijse, 1998; Lamme & Roelfsema, 2000) classified two components of neural responses in V1: an early feedforward component and a later re-entrant feedback component. They hypothesized that the feedforward component represents pre-attentive and unconscious processing, while the feedback component is involved in conscious attentive processing. The early component passes V1 about 40–80 ms after the onset of the visual stimulus. The time course of the later component is not yet clear.

Transcranial magnetic stimulation (TMS) has been used to characterize the time course of processing in striate and circumstriate areas. With this technique, a cortical region can be stimulated through the intact skull. A strong transient electromagnetic field is induced for about 300 µs with a coil placed at the skull. The field penetrates the bone without resistance and in turn induces an electric field within the cortex. This transient electric field induces a pattern of neuronal excitation and inhibition in the network located under the stimulation coil as well as in remote areas (Ruff, Blankenburg, Bjoertomt, Bestmann, Freeman, Haynes, Rees, Josephs, Deichmann, & Driver, 2006). The rather unspecific neural response can interfere with visual processing, acting like a mask (Kammer, Puls, Strasburger, Hill, & Wichmann, 2005a). In the first demonstration of this effect (Amassian, Cracco, Maccabee, Cracco, Rudell, & Eberle, 1989) a letter identification task was used. A string of three letters was flashed on a computer screen. The contrast of the letters was reduced such that subjects were just able to report them correctly under conditions without TMS. A strong TMS pulse over the occipital cortex was applied with a stimulus onset asynchrony (SOA) varied from 0 ms to 200 ms after the letter presentation. An SOA of

100 ms maximally suppressed letter identification down to a recognition rate of zero. Varying the SOA from 0 ms to 200 ms revealed a U-shaped function of the suppression effect that started at about 60 ms and ended at about 120 ms SOA.

In many studies the TMS-induced masking effect has been replicated, with the most effective SOA in a time window of 80–130 ms after the visual stimulus. It shortens with luminance increase of the visual object (Kammer, Puls, Strasburger, Hill, & Wichmann, 2005a), similar to the P100 component in pattern-reversal visual evoked potentials (VEP). Beside this robust effect, several earlier SOA windows with TMS-induced masking effects have been reported. They have been attributed to the early feedforward component of neural activity in V1. In the following I will review the data on this early TMS masking effect.

## Early TMS masking effects

Two different forms of early masking effects have been observed: (i) a broadening of the effective SOA window, (ii) an early distinct SOA peak in addition to the well-known peak around 100 ms. Only the second observation supports the hypothesis of two components of neural responses in V1.

A broadening of the SOA window was first described by Beckers and Hömberg (1991). Using the letter identification task introduced by Amassian et al. (1989), correct response rates dropped at an SOA of 80 and 100 ms for a moderate TMS intensity. Increasing TMS intensity suppressed letter identification already at 40 ms, and to a smaller extent at 60, 80, 100, and 120 ms. In a recent study (Kammer et al., 2005a), we determined contrast thresholds in an object orientation task. We found an effect of TMS intensity at SOAs consistent with Beckers and Hömberg. While the masking effects peaked around 100 ms, a moderate masking occurred at SOAs below 80 ms in three out of four subjects when increasing TMS intensity. For one participant, the moderate effect started with an SOA of zero, i.e. the simultaneous presentation of the visual object and the TMS stimulus. For the other two, even a negative SOA of – 50 ms (TMS before visual stimulus) revealed a moderate masking effect. Similar to Beckers and Hömberg (1991), we did not obtain any evidence for two separate SOA periods but rather observed a broadening of the SOA window.

In contrast to the broadening of the SOA window, two distinct SOA periods with masking effects have been observed in some experiments. Paulus, Korinth, Wischer, & Tergau (1999a, 1999b) presented Gaussian dots with different colors and determined contrast thresholds on color discrimination. TMS masking was most prominent at an SOA of 90 ms with the chromatic dots. In the case of the achromatic controls (darker or brighter than background), two SOA maxima were observed, the first at 30 ms and the second at 90 ms.

In a series of experiments, Corthout et al. (Corthout, Uttl, Walsh, Hallett, & Cowey, 1999; Corthout, Uttl, Juan, Hallett, & Cowey, 2000; Corthout, Hallett, & Cowey, 2002) presented evidence for several SOA periods resulting in a TMS masking effect. They used a letter identification task with five letters. The masking effect at an SOA of 100 ms was obtained in all subjects (Corthout et al., 1999, 2002). Masking periods with negative SOA, i.e. TMS pulse before onset of visual stimulus, were related to a TMS-induced eye blink (Corthout et al., 1999). In some subjects, an early window of TMS masking around 20 ms was observed, which seemed to be independent of the robust SOA effect around 100 ms (Corthout et al., 1999). Unfortunately, this early SOA period could not be reproduced in subsequent experiments (Corthout et al., 2000, 2003), but with each new experiment a new SOA period was identified and named dip0 (induced blink), dip1 (maximum SOA 20 ms), dip2 (maximum SOA 100 ms) and a somewhat cryptic dipX. The four dips have never been observed simultaneously. In the discussion Corthout et al. (2003) offered many explanations for the cryptic finding but systematically rejected any of them ending with the statement “The present study demonstrates the complexity of TMS as a technique to study visual perception.”

Using a two-alternative forced choice vernier discrimination task, one out of three subjects showed a clearly separated early peak of masking SOA with a local maximum at around 40 ms (Kammer, Scharnowski, & Herzog, 2003), comparable to the findings of Paulus et al. (1999a) and Corthout et al. (1999).

## Discussion

Two conclusions can be drawn from the data published so far: (i) Whereas the TMS masking effect at an SOA of 100 ms is robust and reproducible, an early TMS effect seems to be weaker and might only be present in a subgroup of subjects investigated. (ii) Strong TMS pulses seem to increase the duration of the induced masking effect.

Single-pulse TMS not only tells us something about the role of the stimulated cortical region but in addition about the time course of processing within that region. So far, time course data have been interpreted under the assumption that the neuronal effect induced by a TMS pulse emerges without a delay. This is quite plausible since the induced depolarization takes place within one millisecond. However, single cell recordings from a cat’s visual cortex demonstrate that the TMS-induced effects may last for seconds (Moliadze, Zhao, Eysel, & Funke, 2003). The broadening of the critical SOA window observed by Beckers and Hömberg (1991), as well as by Kammer et al. (2005a), indicates that with strong pulses TMS induced cortical effects may last about 40–100 ms.

The observation of TMS masking at two distinct SOA peaks cannot be explained by prolonged network effects of TMS. It supports the concept of two distinct computational processes taking place in V1. Why the earlier window of TMS masking is less reproducible than the latter remains to be clarified. The type of the visual stimulus and the task subjects have to perform might be critical.

Another explanation for the weak reproducibility of two distinct SOA peaks might be the interindividual anatomical variability that is known to be high in the occipital cortex. Possible target sites for the TMS-induced interference in visual tasks are subcortical structures like the optical radiation, striate and/or extrastriate cortex. In my view, it is most plausible that indeed all the three mentioned structures contribute to the TMS effects (Kammer, Puls, Erb, & Grodd, 2005b). The observed interindividual differences in the masking function could stem from anatomical variability. Furthermore, despite an invariant position of the stimulation coil, the target site for the first SOA peak can not be identical with the target site for the second SOA peak. One could speculate that in the subgroup of subjects demonstrating the first peak, V1 is exposed closer to the skull and therefore more vulnerable to TMS. Further experiments with a detailed analysis of individual functional anatomy are required to clarify this issue.

In conclusion, the TMS experiments published so far provide evidence for two distinct visual processes, but the inconsistencies in the data remain to be clarified.
